# Decreased levels of serum platelet-activating factor acetylhydrolase in patients with rheumatic diseases

**DOI:** 10.1080/09629359791758

**Published:** 1997-06

**Authors:** P. Vergne, V. Praloran, R. Treves, Y. Denizot

**Affiliations:** 1 Service de Rhumatologie CHRU Dupuytren 2 rue M. Luther King Limoges 87042 France; 2 Laboratoire d'Hématologie Expérimentale Faculté de Médecine 2 rue Dr. Marcland Limoges 87025 France

## Abstract

PAF is a potent inflammatory compound known to stimulate the release of various cytokines involved in rheumatic diseases. Elevated blood PAF levels are reported in these patients. We report that serum PAF acetylhydrolase activity (AHA) levels are decreased in patients with rheumatoid arthritis or osteoarthritis as compared to healthy controls. Serum and synovial fluid AHA levels were correlated in these patients. The present study suggests the potential role of AHA in controling systemic and/or local PAF levels in patients with rheumatic diseases.


Mediators of Inflammation, **6**, 241–242 (1997)

### Decreased levels of serum platelet-activating factor acetylhydrolase in patients with rheumatic diseases

P. Vergne,[1] V. Praloran,[2] R. Treves[1] and Y. Denizot[2,CA]

[1] Service de Rhumatologie, CHRU Dupuytren, 2 rue M. Luther King, 87042 Limoges, France; and [2]Laboratoire d'Hématologie Expérimentale, Faculté de Médecine, 2 rue Dr. Marcland, 87025 Limoges, France

[CA]Corresponding author
Fax: (+33) 05 55 43 58 01, (+33) 05 55 43 58 66

**PAF is a potent inflammatory compound known to stimulate the release of various cytokines involved in rheumatic diseases. Elevated blood PAF levels are reported in these patients. We report that serum PAF acetylhydrolase activity (AHA) levels are decreased in patients with rheumatoid arthritis or osteoarthritis as compared to healthy controls. Serum and synovial fluid AHA levels were correlated in these patients. The present study suggests the potential role of AHA in controling systemic and/or local PAF levels in patients with rheumatic diseases.**

**Key words:** Acetylhydrolase activity, Osteoarthritis, Rheumatoid arthritis

### Introduction

Platelet-activating factor (PAF), a phospholipid molecule with potent inflammatory activities, is involved in several inflammatory ailments in man.[1] Thus, regulating PAF levels is of importance since elevated concentrations of PAF could result in pathological effects.[1] Blood PAF levels are regulated by an acetylhydrolase activity (AHA) found in plasma and serum.[2] PAF is present in blood and synovial fluid of patients with various arthropathies with higher concentrations in rheumatoid arthritis.[3] It is reported that these patients have higher serum AHA levels than healthy controls,[4] a result that does not fit well with their elevated blood PAF levels. Contradictory results are also reported concerning the correlation between AHA levels in synovial fluid and serum of rheumatoid arthritis patients.[4,5] In order to clarify these points, we have assessed AHA in the serum and synovial fluid of patients with rheumatoid arthritis and osteoarthritis.

### Patients and Methods

Samples were obtained from patients presenting active disease and from healthy individuals according to the Helsinki recommendations. Fifty four patients had a rheumatoid arthritis according to the American Rheumatism Association criteria. The sex ratio (M/F) was 0.5 and the average age was 61.2 years (range 27–75). Twenty six patients had a symptomatic osteoarthritis; the sex ratio in this group was 1 and the average age was 67.7 years (range 45–75). Seventy four healthy individuals served as controls; the sex ratio (M/F) was 2.2 and the average age was 50.7 years (range 20–98). Sera (collected from all patients and controls) and synovial fluids (collected from 12 and 13 patients with rheumatoid arthritis and osteoarthritis, respectively) were stored at −80°C until assay of AHA.

AHA was measured by the degradation of [$^3$H]PAF as previously reported.[6,7] Briefly $1 \times 10^5$ dpm of 1-0-alkyl-2-[$^3$H]acetyl-glycerophosphocholine ([$^3$H]acetyl-PAF, 10 Ci/mmol, NEN), 0.1 mM PAF, HEPES buffer (pH 7.8) in a final volume of 450 μl, and 50 μl diluted serum (1:50 dilution in HEPES buffer) were incubated for 20 min at 37 °C. The reaction was stopped with 100 μl BSA (10%) and 400 μl trichloracetic acid (20%). Samples were centrifuged (1500 × ***g***, 15 min) and supernatants counted in a liquid scintillation counter. Results are expressed as nanomoles PAF degraded per ml of serum or synovial fluid (nmol/min/ml) as means of duplicate determinations. The variation between duplicates was less than 6%.

Differences between groups were assessed by Mann–Whitney U-test. Serum and synovial fluid AHA levels were correlated by linear regression analysis.

© 1997 Rapid Science Publishers 

### Results and Discussion

Serum AHA levels were significantly ($P \leqslant 0.0004$) decreased in patients with rheumatoid arthritis ($49.7 \pm 2.2$ nmol/min/ml) or osteoarthritis ($62.8 \pm 3.2$ nmol/min/ml) as compared to healthy controls ($74.3 \pm 1.9$ nmol/min/ml) (Fig. 1). AHA levels in synovial fluid were not significantly ($P = 0.91$) different in patients with rheumatoid arthritis ($41.8 \pm 5.5$ nmol/ml/min) and osteoarthritis ($44.9 \pm 8.0$ nmol/ml/min). Serum and synovial fluid AHA levels were correlated in patients with rheumatoid arthritis ($r = 0.83$, $P = 0.0016$) and osteoarthritis ($r = 0.57$, $P = 0.04$).

Serum AHA levels in our healthy individuals were similar to previous reports by us and others.[7–11] As already documented,[4,5] we found that AHA levels in synovial fluid were not different in patients with rheumatoid arthritis and osteoarthritis. In our study, AHA levels in synovial fluid were correlated with those in the serum. In contrast to a previous report,[4] we found a significant decrease of serum AHA levels in patients with rheumatic diseases especially in those with rheumatoid arthritis. This result might, in part, explain the elevated circulating blood PAF levels reported in these patients.[3] PAF is a potent inflammatory compound known to stimulate the release of various cytokines (such as IL-1, IL-6 and TNF-α) involved in rheumatoid arthritis.[12,13] Thus, the present study suggests the potential role of AHA in controlling systemic and/or local PAF levels in patients with rheumatic diseases.

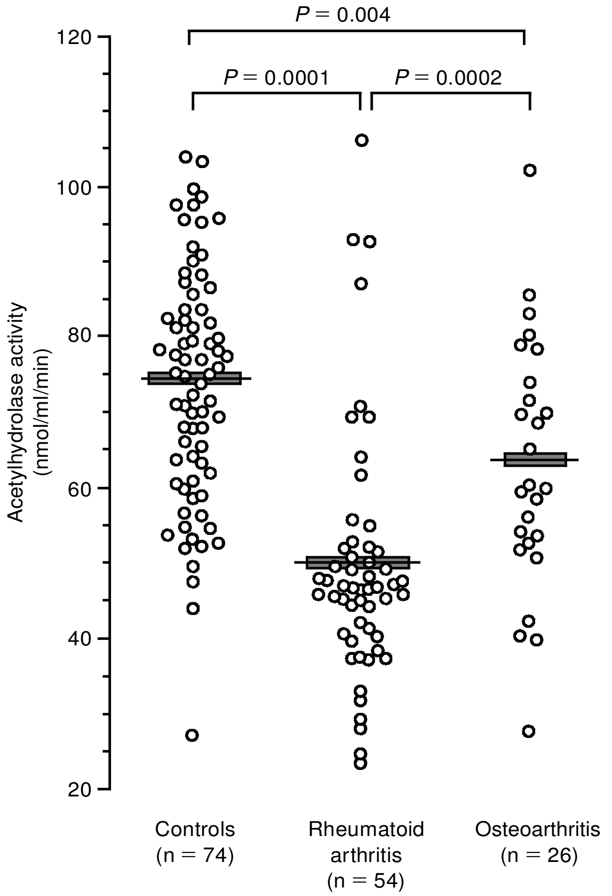

FIG. 1. Serum AHA levels in patients with rheumatic diseases and in healthy individuals. Results are expressed in nmol/min/ml (individual data). Differences were assessed by the Mann–Whitney U-test. The shaded areas represent the mean ± SEM of values.

### References

1. Braquet P, Touqui L, Shen TY, Vargaftig BB. Perspective in platelet-activating factor research. *Pharmacol Rev* 1987; **39**: 97–145.
2. Stafforini DM, Prescott SM, Zimmerman GA, McIntyre TM. Mammalian platelet-activating factor acetylhydrolases. *Biochim Biophys Acta* 1996; **1301**: 161–173.
3. Hilliquin P, Menkes CJ, Laoussadi S, Benveniste J, Arnoux B. Presence of PAF-acether in rheumatic diseases. *Ann Rheum Dis* 1992; **51** 29–31.
4. Dulioust A, Hilliquin P, Menkes CJ, Benveniste J, Arnoux B. Paf-acether acetylhydrolase activity is increased in patients with rheumatic diseases. *Scand J Rheumatol* 1992; **21**: 161–164.
5. Hilliquin P, Houbaba H, Aissa J, Benveniste J, Menkes CJ. Correlations between PAF-acether and tumor necrosis factor in rheumatoid arthritis. *Scand J Rheumatol* 1995; **24**: 169–173.
6. Miwa M, Miyake T, Yamanaka T, *et al.* Characterization of serum platelet-activating factor (PAF) acetylhydrolase. Correlation between deficiency of serum PAF acetylhydrolase and respiratory symptoms in asthmatic children. *J Clin Invest* 1988; **82**: 1983–1991.
7. Nathan N, Denizot Y, Feiss P. Eicosanoid and cytokine levels in plasma of patients during mesenteric infarction. *Med Inflamm* 1997; **6**: 75–77.
8. Pritchard PH, Chonn A, Yeung CCH. The degradation of platelet-activating factor in the plasma of a patient with familial high density lipoprotein deficiency (Tangier Disease). *Blood* 1985; **66**: 1476–1478.
9. Satoh K, Imaizumi TA, Kawamura Y, Yoshida H, Takamatsu S, Mizono S. Activity of platelet-activating factor (PAF) acetylhydrolase in plasma from patients with ischemic cerebrovascular disease. *Prostaglandins* 1988; **35**: 685–698.
10. Denizot Y, Dupuis F, Trimoreau F, Praloran V. Decreased levels of platelet-activating factor in blood of patients with lymphoid and nonlymphoid hematologic malignancies. *Blood* 1995; **85**: 2992–2993.
11. Nathan N, Denizot Y, Huc MC, *et al.* Elevated levels of PAF-acether in blood in patients with type 1 diabetes mellitus. *Diabete Metab* 1992; **18**: 59–62.
12. Arend WP, Dayer JM. Cytokines and cytokine inhibitors or antagonists in rheumatoid arthritis. *Arthritis Rheum* 1990; **33**: 305–315.
13. Bonavida B, Mencia-Huerta JM. Platelet-activating factor and the cytokine network in inflammatory processes. *Clin Rev Allergy* 1995; **12**: 381–395.

**Received 9 April 1997; accepted 10 April 1997**

## References

[PDF_00178] Arend W. P., Dayer J. M. (1990). Cytokines and cytokine inhibitors or antagonists in rheumatoid arthritis.. Arthritis Rheum.

[PDF_00180] Bonavida B., Mencia-Huerta J. M. (1994). Platelet-activating factor and the cytokine network in inflammatory processes.. Clin Rev Allergy.

[PDF_00145] Braquet P., Touqui L., Shen T. Y., Vargaftig B. B. (1987). Perspectives in platelet-activating factor research.. Pharmacol Rev.

[PDF_00172] Denizot Y., Dupuis F., Trimoreau F., Praloran V., Liozon E. (1995). Decreased levels of platelet-activating factor in blood of patients with lymphoid and nonlymphoid hematologic malignancies.. Blood.

[PDF_00152] Dulioust A., Hilliquin P., Menkes C. J., Benveniste J., Arnoux B. (1992). Paf-acether acetylhydrolase activity is increased in patients with rheumatic diseases.. Scand J Rheumatol.

[PDF_00155] Hilliquin P., Houbaba H., Aissa J., Benveniste J., Menkes C. J. (1995). Correlations between PAF-acether and tumor necrosis factor in rheumatoid arthritis. Influence of parenteral corticosteroids.. Scand J Rheumatol.

[PDF_00150] Hilliquin P., Menkes C. J., Laoussadi S., Benveniste J., Arnoux B. (1992). Presence of paf-acether in rheumatic diseases.. Ann Rheum Dis.

[PDF_00158] Miwa M., Miyake T., Yamanaka T., Sugatani J., Suzuki Y., Sakata S., Araki Y., Matsumoto M. (1988). Characterization of serum platelet-activating factor (PAF) acetylhydrolase. Correlation between deficiency of serum PAF acetylhydrolase and respiratory symptoms in asthmatic children.. J Clin Invest.

[PDF_00162] Nathan N., Denizot Y., Feiss P. (1997). Eicosanoid and cytokine levels in plasma of patients during mesenteric infarction.. Mediators Inflamm.

[PDF_00175] Nathan N., Denizot Y., Huc M. C., Claverie C., Laubie B., Benveniste J., Arnoux B. (1992). Elevated levels of paf-acether in blood of patients with type 1 diabetes mellitus.. Diabete Metab.

[PDF_00165] Pritchard P. H., Chonn A., Yeung C. C. (1985). The degradation of platelet-activating factor in the plasma of a patient with familial high density lipoprotein deficiency (Tangier disease).. Blood.

[PDF_00168] Satoh K., Imaizumi T., Kawamura Y., Yoshida H., Takamatsu S., Mizono S., Shoji B., Takamatsu M. (1988). Activity of platelet-activating factor (PAF) acetylhydrolase in plasma from patients with ischemic cerebrovascular disease.. Prostaglandins.

[PDF_00147] Stafforini D. M., Prescott S. M., Zimmerman G. A., McIntyre T. M. (1996). Mammalian platelet-activating factor acetylhydrolases.. Biochim Biophys Acta.

